# Behavioral Psychedelics: Integrating Mind and Behavior to Improve Health and Resilience

**DOI:** 10.3389/fpsyt.2022.821208

**Published:** 2022-03-14

**Authors:** Edmund C. Neuhaus, George M. Slavich

**Affiliations:** ^1^Department of Psychiatry, McLean Hospital/Harvard Medical School, Belmont, MA, United States; ^2^atai Life Sciences, San Diego, CA, United States; ^3^Department of Psychiatry and Biobehavioral Sciences, University of California, Los Angeles, Los Angeles, CA, United States

**Keywords:** psychedelics, psychotherapy, intervention, treatment, guidelines, chronic disease, resilience, behavioral health

## Abstract

One of the most recent and potentially promising advancements in the health sciences has involved the attempted use of psychedelics for treating mental and behavioral health problems, such as anxiety, depression, posttraumatic stress disorder, and addiction. Despite surging scientific and public interest in this work, however, we presently have no standard of care or consensus regarding how best to combine psychotherapy and psychedelics or to assess effectiveness. We discuss these timely issues here through the lens of *behavioral psychedelics*, which we define as the study of psychedelics to foster intentional changes in habits and behaviors to improve health and resilience. Psychedelics may have the potential to reduce chronic disease risk caused by mental and behavioral rigidity. To fully realize this potential, though, we believe the field must establish best practices and guidelines that include how to induce lasting changes in behavior.

Most people living today will die not from communicable diseases but from chronic disease conditions that include heart disease, cancer, diabetes, and Alzheimer's disease, as well as depression, posttraumatic stress disorder (PTSD), and addiction. These conditions reduce quality of life and lead to functional impairment, suicide, and premature death. Indeed, chronic diseases are the leading cause of disability worldwide and yield $3.8 trillion in annual healthcare costs in the United States (1), making them one of our most pressing problems.

To address this public health problem, large sums of money have been poured into genetic studies aimed at elucidating the biological basis of chronic disease risk. Although important, risk for most chronic diseases is determined not primarily by genetics but by behavioral factors such as diet, sleep, exercise, alcohol use, smoking, and acting in ways that engender chronic stress (i.e., stress generation). In a recent analysis of 1.53 million deaths, for example, only 16.4% were attributable to genetics plus shared exposures (2). Therefore, improving public mental and physical health must involve transforming how people behave.

Changing human behavior may sound simple but is exceedingly difficult, especially for behaviors that arise from years of thinking and acting in relatively rigid, routinized ways. One emerging strategy for accomplishing behavior change involves using psychedelic compounds to make the mind more malleable and open (3). A recent demonstration of this potential was the first major randomized, double-blind, placebo-controlled Phase 3 study that investigated the efficacy and safety of 3,4-methylenedioxymethamphetamine (MDMA)-assisted therapy for helping patients work through severe PTSD (4). The treatment improved psychosocial functioning and demonstrated efficacy for reducing PTSD and depressive symptoms. Additionally, a recent Phase 2 clinical trial of the psychedelic compound psilocybin reduced depression when combined with psychotherapy (5).

These data suggest that psychedelics-assisted psychotherapy may be beneficial. Additionally, a recent cost-effectiveness analysis found that MDMA-assisted psychotherapy (vs. standard care) per 1,000 patients may save $103.2 million over 30 years, with costs breaking even after only 3.1 years (6). Despite this good news, however, there is presently no consensus regarding how to best combine psychotherapy and psychedelics. The general psychotherapeutic approach currently employed in trials is non-directive and involves several phases: assessment, preparation, psychedelic experience, and post-dosing integration (3). Looking forward, we believe that further refinement is needed to operationalize and test these components to establish a best-practice standard of care for treating psychiatric, addiction, somatic, and behavioral health problems.

These issues can be addressed by what we herein call *behavioral psychedelics*, or the study of psychedelics to foster intentional changes in habits and behaviors to improve health and resilience. Behavioral psychedelics can realize this potential by developing targeted approaches for therapeutic change that help people achieve enduring functional improvements in self-care, social connection, and family, school, and community responsibilities to help them live the life they desire.

One priority problem for behavioral psychedelics to tackle concerns reducing psychological rigidity and dysfunctional behaviors in chronic illness. Many such illnesses involve pervasive, maladaptive behavioral patterns that give rise to feeling hopeless, ineffective, and disconnected from oneself and others. Psychedelic compounds can help break this rigidity by inducing time-limited neuroplasticity that enhances psychological flexibility, expands insight, and makes individuals open to life changes (7). This change of mind, however, does not necessarily mean that patients *know* how to change their entrenched patterns. Behavioral psychedelics can address this by providing a structured, supportive framework to help patients *learn how to live differently* by replacing dysfunctional attitudes and behaviors with new skills that support healthier living.

To achieve this, the behavioral psychedelics model includes the self-reinforcing elements of planning, action, and motivation that work synergistically with the psycho-neuro flexibility induced by psychedelics to create positive change. These changes will ideally soon become self-sustaining as individuals learn and use their new skills and experience success, leading to improved mood and greater self-efficacy (8). On the path to establishing a standard of care, we believe providers should work collaboratively with patients during the preparation phase to critically analyze their behavioral patterns and develop a treatment plan and goals that set priorities for a new life course. The post-dosing phase, in turn, is the opportune period of neuroplasticity and newfound psychological flexibility from the psychedelic experience that can enable people to begin shifting their life perspective and enact new behaviors. A structured approach guides behavior and habit changes and provides concrete ways for an individual to monitor progress and adjust goals, on their terms, to improve health, resilience, and connection—all in stark contrast to living with chronic disease (see Figure 1).

**Figure 1:**
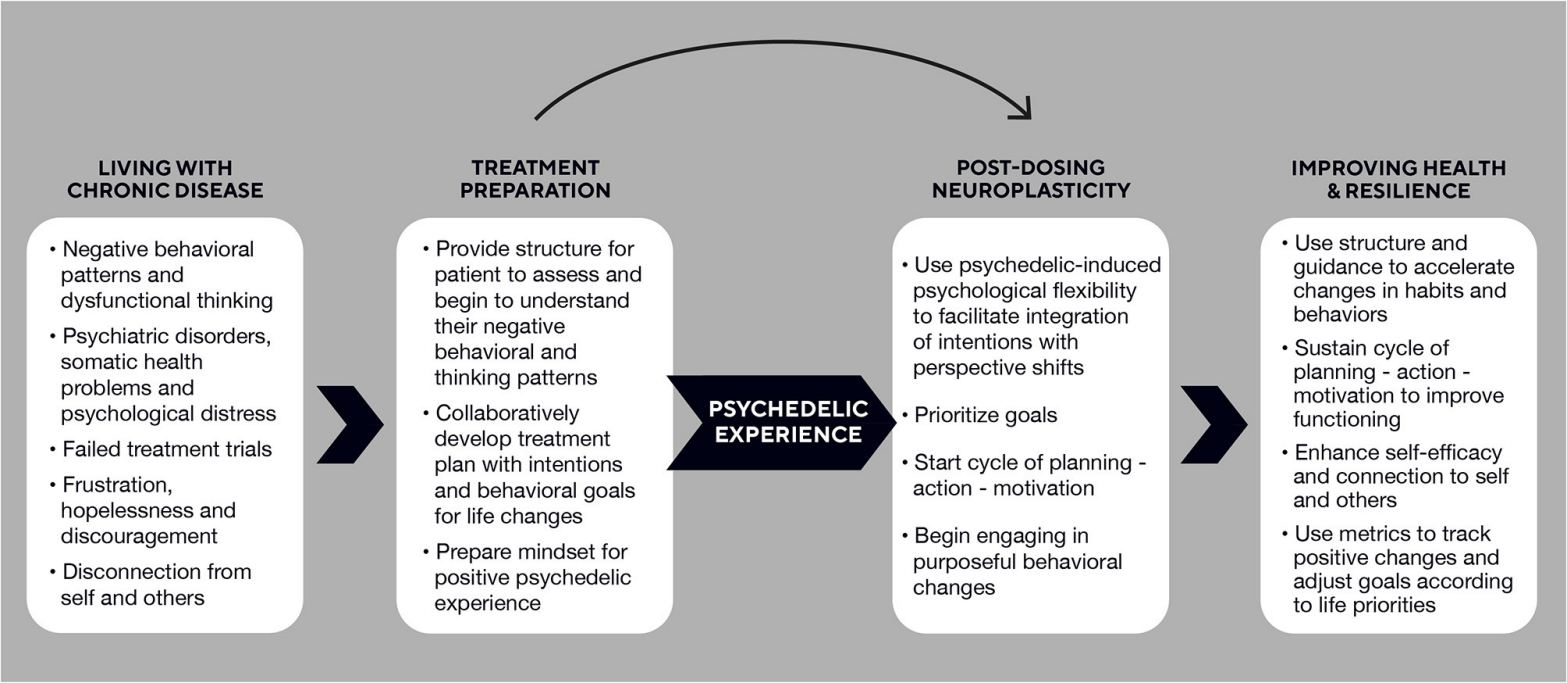
Path of Behavioral Psychedelics to foster intentional changes in behaviors and habits to improve health and resilience.

Psychedelics show promise for enhancing efficacy over conventional standards of care. Yet, with few data presently available from high-quality RCTs, a main challenge is to empirically validate combination treatments for addressing treatment-resistant disorders. Several related issues also require attention. What are the criteria for optimizing the match of psychedelic compounds for different diagnoses? What approaches (e.g., non-directive vs. directive, individual and group therapies, digital platforms) are most useful for helping people realize their goals, in their unique life context? What role do placebo effects play? And, what psychosocial, biological, and behavioral outcomes should be measured to establish the efficacy of—and mechanisms of change underlying—psychedelic-related improvements in health and behavior?

More broadly, with the rapid changes induced by psychedelics, how do we help individuals adjust to their new, healthier selves as they return to social environments that have not changed (at best) or are dysfunctional or harmful (at worst)? How do we help individuals stay engaged in treatment when there is a tendency to leave? Finally, how can we maximize the accessibility and scalability of these approaches to address the magnitude of the chronic disease crisis?

Psychedelic compounds have the potential to turbocharge the process of transforming the mind, and the race to realize their benefits is in full swing. To maximize these benefits, we believe this work should include behavior as a treatment target with measurable treatment metrics to establish best practices and guidelines. Focusing on behavior will lead to more rigorous research on psychedelics but, most importantly, it will also help change what is most responsible for maintaining chronic disease.

## Data Availability Statement

The original contributions presented in the study are included in the article/supplementary material, further inquiries can be directed to the corresponding author/s.

## Author Contributions

All authors listed have made a substantial, direct, and intellectual contribution to the work and approved it for publication.

## Funding

EN is an employee of atai Life Sciences. GS was supported by grant #OPR21101 from the California Initiative to Advance Precision Medicine and by contract #21-10317 from the Office of the California Surgeon General and California Department of Health Services, which supports the UCLA-UCSF ACEs Aware Family Resilience Network. These organizations had no role in the planning, writing, editing, or reviewing of this article or in the decision to submit this article for publication.

## Conflict of Interest

EN is Senior Director of Psychology for atai Life Sciences, which produces psychedelic compounds. GS has consulted for atai Life Sciences.

## Publisher's Note

All claims expressed in this article are solely those of the authors and do not necessarily represent those of their affiliated organizations, or those of the publisher, the editors and the reviewers. Any product that may be evaluated in this article, or claim that may be made by its manufacturer, is not guaranteed or endorsed by the publisher.
